# A Case of Lead Poisoning due to a Mixture of Talisman Ash

**DOI:** 10.1186/2052-4374-25-37

**Published:** 2013-11-28

**Authors:** Han Hui Ye, Jae Uk Jeong, Nak Joon Baek, Chang Yul Choi, Man Joong Jeon, Joon Sakong

**Affiliations:** 1 Department of Occupational and Environmental Medicine, Yeungnam University Hospital, 317-1, Daemyungdong, Namgu, Daegu 705-717, Republic of Korea

**Keywords:** Lead poisoning, Talisman, Cinnabar, Acute hepatitis

## Abstract

**Background:**

Lead is a metal that has no biological function useful for the human body. In Korea, non-occupational exposure to lead has mostly occurred through taking oriental medicine. However, in this paper we report a case of lead poisoning caused by ingesting talisman material.

**Case presentation:**

A 16-year-old male patient complained of severe abdominal pain after taking cinnabar, a talisman material. He was diagnosed with lead poisoning accompanied by acute hepatitis. We confirmed that the cinnabar the patient took contained about 10% elemental lead. After symptom management, the patients’ symptoms, liver function test results, and blood lead concentration level improved.

**Conclusion:**

Lead poisoning can be accompanied by hepatitis, although rarely. As we have confirmed that cinnabar as a talisman material is harmful to the human body, measures to prevent its misuse are needed.

## Background

Lead is the most commonly used non-ferrous metal [1]. Humans have refined lead since 4000BC and have used it for making various objects, including covering materials, alloys, antiknock agents, the pole plates of electrical batteries, crystal glass, vinyl chloride stabilizers, pigments (paint, drawing tools, coloring of rubber, pottery glaze), pesticides, insecticides, radiation shields, and bullets [2]. Historical records of lead poisoning include Hippocrates’ report of abdominal colic pain caused by lead poisoning in 370BC; lead pallor, constipation, lead colic, and lead palsy described by Nicander in 2BC [3-5]; and lead poisoning at an illegal brewery reported in the 1700s [6,7]. After the 1920s, cases of children’s pica from lead-containing paint were reported [8].

Lead exposure to the human body usually occurs by inhalation of lead dust and fumes through the respiratory tract or digestive system [5]. It has been reported that 37-70% (on average 60%) of the ingested amount of lead is absorbed on an empty stomach; 4-21% in the case of soluble lead salt ingested with food; and 15-20% on average [9]. The absorbed lead is combined with red blood cells and distributed to diverse soft tissues such as the brain, liver, bone marrow, and testes, then stored in the bone [10]. Lead is a metal that is known to have no biological function useful to the human body and accumulates in the body in proportion to the exposure amount, affecting the erythropoietin system, digestive system, central and peripheral neurologic system, muscles, kidney, cardiovascular system, and reproductive system [11]. In addition, while there are many animal test results showing that lead exposure damages liver function, such cases in the human population are very rare [12].

Lead exposure is categorized into occupational and non-occupational exposure [8]. Occupational exposure includes the processes of metal refining, storage battery manufacturing, soldering, glass manufacturing, and glaze production [13]. Non-occupational exposure includes intake through the water supply through lead-containing water pipes, inhalation of paint and gasoline fumes, pollution of soil, burning of newspaper, tin cans, and cookware [8]. In Asian countries including Korea, cases of lead poisoning by oriental medicine are also frequently reported. Recently, such cases have been reported by Asian immigrants in the United States and Europe as well [14]. Fung et al. [15] reported a lead poisoning case in which a female patient complained of general myalgia and anemia after taking self-manufactured oriental medicine for her acne. Oh et al. [2] and Choi et al. [12] also reported cases of lead poisoning caused by the administration of oriental medicine. However, to date, no lead poisoning case caused by the administration of the mixture of cinnabar, talisman material, and talisman ashes as manufactured by fortune-telling shops has been reported. The red letters of a talisman are produced by grinding cinnabar. Although pure cinnabar is mostly composed of mercury, due to its high cost, cinnabar that contains impurities is available on the market.

In this study, along with a literature review, we present a case of lead poisoning accompanied by acute hepatitis in which a patient complained of abdominal colic pain after ingesting a mixture of talisman material and talisman ashes.

## Case presentation

**Patient:** 16-year-old male. The patient signed an informed consent statement. This study was conducted in compliance with the international Declaration of Helsinki. The institutional review board of our university hospital approved the study protocol.

**Chief complaint:** abdominal pain

**Past medical history:** no specific findings

**Occupation:** high school student

**Family history:** no specific findings

### Current medical history

The patient’s father received cinnabar and a mixture of talisman ashes (approximately 6 g) following the recommendation of the owner of a fortune-telling shop in the city of Daegu, and had the patient take a total of five doses of the mixture from April 7 to 11, 2013, one dose per day with water. Later, on April 14, the patient experienced discomfort in the upper abdominal quadrant and several episodes of vomiting and diarrhea. After receiving symptom management for enteritis at a local clinic, the patient’s symptoms improved in three more days. However, on April 21, beginning at early dawn, the patient experienced increasing pain in the whole abdomen—mainly in the left lower quadrant—which was squeezing and piercing in nature, along with several bouts of vomiting and diarrhea. He then visited a regional hospital and underwent blood testing. His test results included the following: AST 250 U/L; ALT 1130 U/L; γ-GTP 244 U/L; and total bilirubin 3.0 mg/dL (direct bilirubin 0.7 mg/dL). The result of an abdominal sonogram was normal. Abdominal CT results showed hepatosplenomegaly, suspected lymphadenopathy in the right lower quadrant, and suspected presence of body fluid in the pelvic cavity. Based on the results, the patient was diagnosed with acute hepatitis. Later, the patient was transferred to C University Hospital as requested by his parents and was hospitalized and treated for another week. During hospitalization, tests for diagnosing his abdominal pain and hepatitis were conducted. Among the imaging tests, a chest X-ray, abdominal X-ray, and abdominal CT scan showed normal results. An abdominal sonogram showed liver function impairment and slight splenomegaly. A liver-spleen scan confirmed diffuse parenchymal liver disease. The results of a sigmoidoscopic examination were normal. The lab test results were as follows: no specific findings from a Widal test; HAV IgG Ab/IgM Ab (-/-); HBs Ag/Ab (-/+); Anti-HCV (-); VDRL non-reactive; HIV Ab (-); CMV IgM (-); HSV IgM (-); EBV EA-IgM (-); and EBV (EBNA) IgM (-). Moreover, there were no specific findings from the tests for typhoid fever, viral hepatitis, autoimmune hepatitis, or Wilson’s disease, as shown by anti-mitochondrial Ab (-), anti-smooth muscle Ab (-), liver/kidney microsomal Ab (-), Rheumatoid factor < 5.0, ANCA (-), FANA (-), autoimmune target test (AIT) (-), ceruloplasmin 19 mg/dL, copper 75.1 μg/dL, u-copper 22.5 μg/day, IgG 840 mg/dL, IgA 267.4 uIU/ml, IgM 65.8 uIU/ml, IgE 169 uIU/ml, and IgD 3.6 mg/dL. The patient’s condition was observed while analgesia and hepatotonics were administered. During the hospitalization, the hemoglobin fell to 11.5 g/dL, and the serum iron was 160.6 μg/dL, TIBC 256 μg/dL, transferrin 182 mg/dL, and ferritin 508.7 ng/mL.

**Figure 1:**
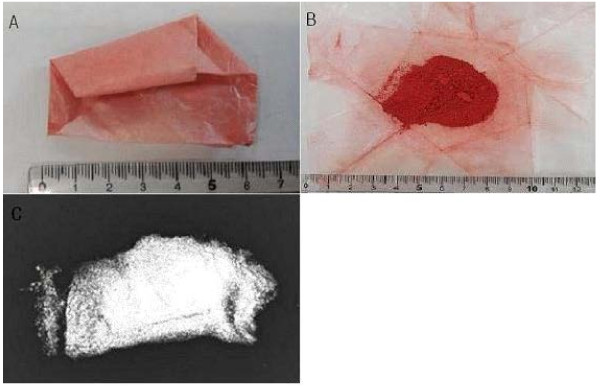
**Cinnabar patient had taken with talisman ash. (A, B)** Cinnabar powder **(C)** A simple X-ray of cinnabar powder.

On April 30, he was discharged from the hospital, as his abdominal pain had been relieved and his liver function test results had improved (Table 1). During follow-up at the outpatient clinic of C University Hospital, the same pattern of abdominal pain as occurred initially recurred in the left side of the abdomen on May 5, and he was hospitalized again. An abdominal CT scan was performed as a follow-up measure, and it showed normal findings. The next day, in order to identify the cause of the continued abdominal pain, the patient was transferred to the Department of Pediatrics of Y University Hospital, per the patient and his parents’ request. During the evaluation, the patient was referred to the Department of Occupational and Environmental Medicine, and after an in-depth interview, it was determined that the patient had taken talisman ashes and cinnabar, a talisman material (Figure 1), and the patient was confirmed to have lead poisoning associated with the administration of that material.

**Table 1:** Laboratory findings of the study subject

**Findings**	**Date**										
	**4/21**	**4/22**	**4/25**	**4/29**	**5/2**	**5/5**	**5/6**	**5/10**	**5/20**	**6/13**	**7/18**
Hb (g/dL)	13.3	13.6	13.2	11.7	11.5	11.8	12.5	11.2	12.6	13.8	15.2
Hct (%)	41.1	40.5	39.1	35.9	35.4	36.0	37.3	32.4	36.9	40.4	43.5
MVC (fL)	91.7	91.3	91.3	92.5	92.9	92.1	-		-	-	-
MCH (pg)	29.7	30.7	30.9	30.3	30.1	30.1	-	-	-	-	-
WBC (/mm^3^)	6000	6600	5800	5100	5600	7000	5900	6530	5570	5770	5250
Segment (%)	59.3	76.3	55.5	58.2	56.3	52.2	-	-	-	-	-
Lymphocyte (%)	29.0	16.5	30.2	30.3	33.9	38.4	-	-	-	-	-
Eosinophil (%)	-	0.1	1.7	1.8	1.8	1.8	-	-	-	-	-
Platelet (K/mm^3^)	210	181	183	205	222	198	257	218	229	202	230
Reticulocyte (%)	-	-	-	-	-	-	5.6	7.0	5.4	3.7	2.1
BUN (mg/dL)	13.1	9.1	17.5	21.9	21.1	14.9	17.5	10.2	12.5	-	-
Cr (mg/dL)	1.0	0.8	0.9	0.8	1.0	0.8	0.9	1.0	0.9	-	-
T-bil (mg/dL)	3.0	4.0	5.1	2.3	1.6	1.5	3.4	1.2	1.5	-	-
D-bil (mg/dL)	0.7	-	-	0.7	0.5	-	1.3	0.6	0.6	-	-
AST (IU/L)	250	236	76	36	36	49	32	29	20	-	-
ALT (IU/L)	1130	844	368	112	64	61	41	36	29	-	-
ALP (IU/L)	175	421	375	274	236	237	115	87	67	-	-
γ-GTP (IU/L)	244	209	187	145	119	112	-	-	-	-	-
Protein (g/dL)	7.5	6.9		6.5	6.5	6.7	-	-	-	-	-
Albumin (g/dL)	4.5	4.4	4.3	4.2	4.3	4.5	-	-	-	-	-
Amylase (mEq/L)	69	58				86	-	-	-	-	-
ESR (mm/hr)	5	3	5	-	-	-	-	-	-	-	-
CRP (mg/dL)	0.1	4.8	0.8	-	-	-	-	-	-	-	-
CK (IU/L)	-	1495	-	-	-	113	-	-	-	-	-
PT (sec)	-	13.3	13.2	13.4	13.2	13.5	-	-	-	-	-
aPTT (sec)	-	37.2	37.6	35.5	33.0	35.6	-	-	-	-	-

*Hb* Hemoglobin; *Hct* Hematocrit; *MVC* Mean corpuscular volume; *MCH* Mean corpuscular hemoglobin; *WBC* White blood cells; *BUN* Blood urea nitrogen; *Cr* Creatinine; *T-bil*, Total bilirubin; *D-bil* Direct bilirubin; *AST* Aspartate transaminase; *ALT* Alanin transaminase; *ALP* Alkaline phophatase; *γ-GTP* γ-glutamyl transpeptidase; *ESR* Erythrocyte sedimentation rate; *CRP* C-reactive protein; *CK* Creatine phosphokinase; *PT* Prothrombin time; *aPTT* activated partial thromboplastin time.

### Health assessment

At the time of the Y University Hospital intake, the patient’s blood pressure was 120/82 mmHg, heart rate 69 bpm, respiration rate 19/min, body temperature 36.6°C, and he was alert, with no signs of acute illness, conjunctival pallor, or icteric sclera. No abnormality was noted in the heart and lung sounds at auscultation. The abdomen was soft, and there was no distention or palpable mass. Tenderness was present in the left lower quadrant. There was no edema or petechiae in any of the four extremities. There were no specific findings in the neurologic exams.

### Test results

**Figure 2:**
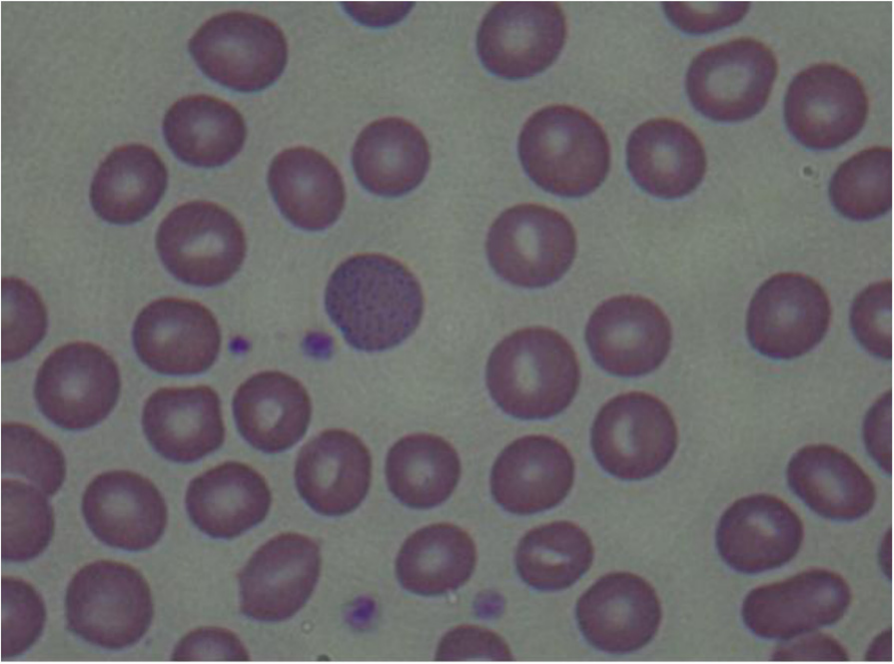
Basophilic stippling of red cells can be noted in a peripheral blood smear.

At the time of hospitalization in Y University Hospital, the results of tests conducted in the Department of Pediatrics showed the following: Hb 12.5 g/dL, Hct 37.3%, MCV 87.4 fL, MCH 29.3 pg, WBC 5900/μL, platelets 257 k/μL, and reticulocytes 5.6%. A peripheral blood smear examination showed normocytic-normochromic anemia and basophilic stippling of the red cells (Figure 2), heptoglobin of 61 mg/dL, a negative Coombs test, additional blood lead concentration of 58.1 μg/dL, zinc protoporphyrin (ZPP) 43.6 μg/dL, and 24-h urine δ-aminolevulinic acid (δ-ALA) 15.1 mg/L. The analysis of the metal ingredients of the cinnabar received from the fortune-telling shop showed 99,636.5 ppm of lead, 166,567.2 ppm of mercury, and 54408.7 ppm of cadmium. No other metal ingredients were found. Later, the patient’s blood cadmium, blood mercury, and urinary mercury concentration were checked, and found to be 0.853 μg/L, 1.942 μg/L, and 3.737 μg/L, respectively. In the meantime, some of the test results showed ongoing improvement compared to the previous test results, including total bilirubin 3.4 mg/dL, AST 32 IU/L, ALT 41 IU/L, and γ-GTP 115 IU/L.

### Treatment and progress

The patient visited our hospital three weeks after the onset of symptoms and was diagnosed with lead poisoning. As the patient had moderate abdominal discomfort and intermittent piercing pain in the left lower quadrant without other specific symptoms at the time of the diagnosis, he was only administered analgesics for abdominal pain. Three days after hospitalization, his symptoms improved, and the patient’s condition was observed without additional pharmacotherapy. He was discharged on the seventh day after intake. Based on monitoring by follow-up tests at the outpatient clinic, his abdominal pain did not recur, nor were there any other notable symptoms. The peripheral blood smear examination conducted on June 13 showed no basophilic stippling of red cells, and the hemoglobin and reticulocyte count improved continuously (Table 1). At the 3 months of follow-up observation, the blood lead concentration was found to have decreased, while the blood ZPP had increased at the 2-month follow-up but had decreased again at the 3-month follow-up (Table 2). The patient is undergoing follow-up on a monthly basis.

**Table 2:** Laboratory findings related to lead

**Findings**	**Date**				
	**5/10**	**5/20**	**6/13**	**7/18**	**8/22**
Pb (μg/dL)	58.1	61.1	58.6	54.7	44.0
ZPP (μg/dL)	43.6		89.4	105.8	71.2
δ-ALA (24-hr urine) (mg/L)	15.1				

*ZPP* zinc protoporphyrin; *δ-ALA* δ-aminolevulinic acid.

## Conclusion

Occupational lead poisoning is significantly decreasing thanks to the enhanced understanding of preventive medicine and occupational health since the 1980s. With regard to non-occupational lead poisoning, due to various policies including mandating the use of lead-free gasoline, banning of the use of paint containing lead, and encouraging the use of lead-free soldered food cans in the U.S. after the mid-1970s, the blood lead concentration of the general public fell as well [8]. In Korea also, since the 1980s, due to the gradual decrease in the lead concentration in the air by the use of lead-free gasoline, use of lead-free paint pigment, and the increased health consciousness of the public, the exposure to lead-contaminated water and food has decreased. As a result, the lead concentration of the general population is not higher than that of many advanced and developing countries [5]. However, unlike in the United States and Europe, in East Asian countries, including Korea, there are many cases of lead poisoning caused by health supplements [8]. The case presented in this paper was caused by ingesting a mixture of talisman material—manufactured and supplied by a fortune-telling shop—and talisman ashes, which has never before been identified as a causative factor for lead poisoning. Unlike cases that have occurred from taking oriental medicine, this case occurred from the short-term administration of a highly concentrated lead-containing substance.

Cinnabar is a natural mineral, in which the main ingredient is mercuric sulfide (HgS). It is also called Jin-sa, Ju-sa, Dan-sa, or Gwangmyeong-sa. It has a red shade and its pure form contains 86.2% mercury. It is also used as a pigment [16]. In fortune-telling shops, it is used to write letters on talismans. In the case presented here, the talisman powder contained 166567.2 ppm of mercury and 99636.5 ppm of lead, the latter of which is a higher concentration of lead than that in pure cinnabar. In oriental medicine, cinnabar is known to have an anti-anxiety and sedative effect [17], and in this case it was used in hopes of the child’s health and bright future.

Symptoms of lead poisoning include changes in the neurologic system, gastrointestinal system, erythropoietin system, muscles, kidneys, cardiovascular system, and reproductive system [11]. Depending on the blood lead concentration, serious neurologic damage such as acute encephalopathy occur at ≥ 80 μg/dL and digestive symptoms such as abdominal pain, constipation, and damage to the erythropoietin system including anemia occur at ≥ 60 μg/dL, and mild neurologic symptoms, cardiovascular symptoms, and kidney function damage at ≥ 30 μg/dL [9]. However, various spectra of such clinical symptoms and signs according to different blood lead concentrations are common in low-concentration chronic lead poisoning and may not correspond to the symptoms and signs of acute lead exposure [18]. In this case, the chief complaint was squeezing pain in the left lower quadrant. In lead poisoning, a common location of abdominal pain is the lower abdomen, intermittently squeezing in nature, and tenesmus also typically occurs [9]. Such symptoms are thought to be caused by the interaction between calcium and lead in the smooth muscle, and intravenous injection of calcium leads to temporary relief [9].

Bone marrow is the most sensitive target organ in lead poisoning. Anemia in lead poisoning occurs by the inhibition of aminolevulinic acid dehydratase (δ-ALA-D)—a heme synthesis enzyme that generates porphobilinogen by conjugating δ-levulinic acid—and ferrochelatase—which combines Fe^2+^ and protoporphyrin IX—to disturb heme synthesis; interfering with functions of the cell membrane and absorption of iron, thereby causing hemolysis; inhibiting various enzymes needed in forming red blood cells; and inhibiting the formation of α and β globin of hemoglobin [19-21].

Basophilic stippled erythrocytes are a typical manifestation of lead poisoning, though not specific to it. For example, it can be observed in hematologic diseases such as thalassemia, vitamin B12 deficiency, pyrimidine 5′ nucleotidase deficiency, or after exposure of the hematopoietic system to harmful substances such as aniline, arsenic, or benzene [21,22].

Lead poisoning and iron deficiency inhibit heme production. In such cases, the heme precursor of protophorphyrin IX creates ZPP, which binds to zinc ions instead of iron ions. Thus, ZPP reflects the lead levels in the bone marrow during cell formation and the ZPP exists as long as the red blood cells remain alive. ZPP levels can, therefore, indicate changes in blood lead levels. In the case presented here, the blood lead levels were found to have decreased in the 2-month follow-up test, while the blood ZPP had increased at month 2 but decreased again by the third month.

The Centers for Disease Control and Prevention (CDC) [23] provides recommended guidelines for the application of chelation therapy for lead poisoning according to the blood lead concentration: For children, application of the therapy is limited to cases with a blood lead concentration ≥ 45 μg/dL; for adults, the therapy is recommended for cases with a blood lead concentration ≥ 100 μg/dL, as symptoms related to the brain or brain dysfunction such as convulsions can accompany these concentration levels. Furthermore, the guideline advises that the use of the therapy should be strongly considered for cases with a blood lead concentration 80-99 μg/dL, and it should be considered selectively when signs and symptoms caused by lead poisoning are present if the blood lead concentration is 50-79 μg/dL [10]. In the case presented here, the patient was asymptomatic by the time he presented to the Occupational and Environmental Medicine Department, his height was 171 cm and weight was 60 kg, and he was being continuously monitored.

Although lead is not known to cause liver toxicity, some cases of lead poisoning accompanied by hepatitis have been reported. Outside Korea, Beattie et al. [24] reported that four patients including teens and those in their 20s who intravenously injected a solution of lead, opium, and water tested negative for hepatitis B antigen, but developed acute hepatitis and fully recovered after chelation therapy. The liver tissue test showed that the lead concentration inside the tissue was 35 times higher than that of occupational lead poisoning. Verheij et al. [25] reported a case in which a 40-year-old Iranian man with multiple sclerosis and no history of drinking who had taken opium on an ongoing basis to control his pain developed lead poisoning due to lead in the opium. The patient had gastrointestinal and hematological symptoms with increased levels in liver function test results (AST 66 IU/L, ALT 92 IU/L, and γ-GTP 729 IU/L) and findings of hepatitis from a liver tissue test. According to the report, the liver function test results improved during the course of chelation therapy. Ibrahim et al. [26] also reported a case in which lead poisoning was accompanied by hepatitis after the administration of oriental medicine. In Korea, Hwangbo et al. [14] reported that the blood lead concentration had a significant influence on GOT from a study of 274 workers exposed to lead. Kim et al. [8] reviewed 45 cases of lead poisoning occurring over the course of 30 years in Korea and reported that the AST was increased in 17 cases and ALT increased in 16 cases, with an average level of 45 ± 14.7 IU/L and 63.4 ± 29.2 IU/L, respectively.

We can conclude that the diagnosis of lead poisoning was delayed in the case presented here due to the following: loose evaluation regarding the cinnabar administration during the initial interview; no impression that may have raised suspicion of lead poisoning other than abdominal pain, based on the symptoms and physical exams; and evaluation of the findings of hepatitis during the imaging diagnostic and lab tests.

This case demonstrated that causes of non-occupational lead poisoning other than oriental medicine or health supplements can include cinnabar and talisman ashes. It also showed a rare incident in which lead poisoning was accompanied not only by the common symptoms of abdominal pain and anemia but also hepatitis. Therefore, medical institutions should conduct more thorough interviews at the initial assessment and consider various possibilities for the cause of symptoms. Furthermore, evaluation of and attention to the misuse of currently available substances from the public health perspective are necessary.

## Competing interests

The authors report no conflict of interests.

## Authors’ contributions

HHY study concept and design, drafting of the manuscript. JUJ analysis of data. NJB analysis of data. CYC analysis of data. MJJ technical support. JS critical revision of the manuscript. All authors read and approved the final manuscript.
